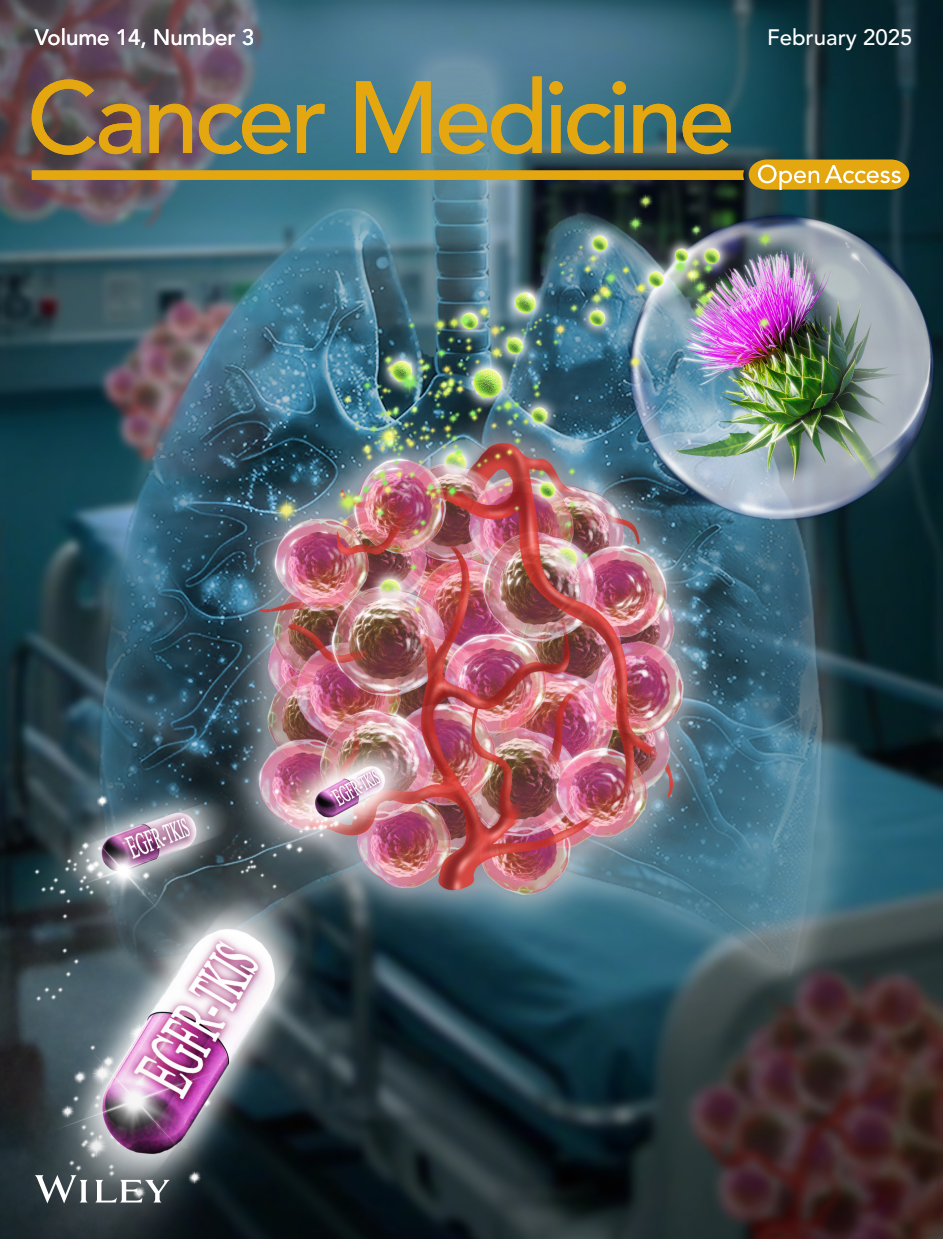# Cover Image

**DOI:** 10.1002/cam4.70760

**Published:** 2025-03-17

**Authors:** Xiaocen Wang

## Abstract

The cover image is based on the article *The Effects of Silibinin Combined With EGFR‐TKIs in the Treatment of NSCLC* by Xiaocen Wang et al., https://doi.org/10.1002/cam4.70643.